# Extremely Rare Polymorphisms in *Saccharomyces cerevisiae* Allow Inference of the Mutational Spectrum

**DOI:** 10.1371/journal.pgen.1006455

**Published:** 2017-01-03

**Authors:** Yuan O. Zhu, Gavin Sherlock, Dmitri A. Petrov

**Affiliations:** 1 Department of Genetics, Stanford University, Stanford, CA, United States of America; 2 Department of Biology, Stanford University, Stanford, CA, United States of America; 3 Genome Institute of Singapore, Singapore; Brigham and Women's Hospital, Harvard Medical School, UNITED STATES

## Abstract

The characterization of mutational spectra is usually carried out in one of three ways–by direct observation through mutation accumulation (MA) experiments, through parent-offspring sequencing, or by indirect inference from sequence data. Direct observations of spontaneous mutations with MA experiments are limited, given (i) the rarity of spontaneous mutations, (ii) applicability only to laboratory model species with short generation times, and (iii) the possibility that mutational spectra under lab conditions might be different from those observed in nature. Trio sequencing is an elegant solution, but it is not applicable in all organisms. Indirect inference, usually from divergence data, faces no such technical limitations, but rely upon critical assumptions regarding the strength of natural selection that are likely to be violated. Ideally, new mutational events would be directly observed before the biased filter of selection, and without the technical limitations common to lab experiments. One approach is to identify very young mutations from population sequencing data. Here we do so by leveraging two characteristics common to all new mutations—new mutations are necessarily rare in the population, and absent in the genomes of immediate relatives. From 132 clinical yeast strains, we were able to identify 1,425 putatively new mutations and show that they exhibit extremely low signatures of selection, as well as display a mutational spectrum that is similar to that identified by a large scale MA experiment. We verify that population sequencing data are a potential wealth of information for inferring mutational spectra, and should be considered for analysis where MA experiments are infeasible or especially tedious.

## Author Summary

The mutational spectrum is central to our understanding of molecular evolution. However, mutational spectra are difficult to study because spontaneous mutations are rare, difficult to observe, and a large number of events is required to detect subtle differences between mutational bias, selection and selection like forces. The possibility of estimating mutational spectra from population polymorphism data, with neither the need for tedious experiments nor the restrictions and biases of lab conditions, is a crucial step in overcoming such difficulties. We show that with sufficiently broad population sequencing and proper identification of young polymorphisms, it is possible to recapitulate the experimental yeast mutation spectrum. This holds implications for future applications to all species where population sequencing is possible.

## Introduction

Knowledge of the mutational spectrum is central to the study of molecular evolution. However, mutational spectra are difficult to characterize because spontaneous mutations are scarce and thus rarely observed in large enough numbers for precise measurements. In addition, mutational spectra vary across species, between individuals, and across genomic segments, placing a demand for methods that can identify a large set of mutational events genome-wide, while remaining applicable to a wide range of species.

One direct approach to the study of spontaneous mutations on a genome-wide scale is through mutation accumulation (MA) experiments. MA experiments allow the accumulation of mutations under minimal selection conditions in a controlled lab environment, usually over many generations [1–4]. If following individual clonal lineages is not feasible, minimal selection conditions are usually achieved in unicellular cultures through repeated extreme bottlenecks, sometimes down to a single individual, such as in *Saccharomyces cerevisiae* [5–13], *Dictyostelium discoideum* [14], *Arabidopsis thaliana* [1], and *Chlamydomonas reinhardtii* [15,16]. It can also be achieved through generations of inbreeding in species such as *Drosophila melanogaster* [17–19], or rhabditid nematods [20]. The final progeny are then sequenced and compared to the starting ancestor to identify *de novo* mutations that occurred within the span of the experiment. The throughput of this process has been greatly aided by recent advances in next generation sequencing, and MA experiments have thus provided significant insights into overall mutation rates, relative frequencies of mutation classes, mutational biases, and repair pathways.

While powerful, MA experiments face certain limitations that cannot be easily rectified. One limitation is technical. Many species cannot be considered for lab studies due to space, life span, ecological, or ethical limitations, if they can be maintained under lab conditions at all. The other limitation is theoretical. Genome stability can be dependent upon environmental factors and life cycle stages [21–23]. For many organisms, including the majority of microbes, such parameters are difficult to characterize. The complex habitats of ‘wild’ populations are thus important but unknown, and therefore cannot be replicated in the lab. In addition, a complex network of genes and pathways regulate DNA repair. Differences in genes involved in DNA fidelity-associated pathways may result in the mutation spectrum varying across sub-populations or even individual strains. As MA experiments usually involve less than a handful of genomic backgrounds that are extremely well adapted to a lab environment, it is possible that they are not representative of the mutational patterns in the species as a whole.

In addition, most MA experiments utilize a relatively small number of lines that are allowed to accumulate relatively large number of mutations for a fairly long period of time. While it is possible to shorten MA experiments, this is often accomplished through the use of mismatch-repair (MMR) impaired strains that accumulate mutations at an artificially fast rate. Such experiments are used to survey large numbers of mutations in a short period of time in a fashion that is specific to the MMR pathway affected. For example, recent work on conditional or complete MMR defect [10, 24–26], nucleotide pool imbalance [27], and replicative polymerase variants [9,13] has made use of such systems. These experiments are powerful but extremely specific means of probing the DNA replication and repair system, and all mentions of MA experiments in the rest of this paper do not specifically refer to MMR based studies.

In regular MA experiments, where the aim is to study ‘natural’ mutations spectrum, only ‘wild-type’ strains are used. For such studies, the MA approach is certainly economical, in that the sequence of a single genome can reveal the presence of a large number of mutations. But the savings come with the cost of two possible sources of bias. First, the MA lines lose fitness as they accumulate mutations and less fit lines might have a very different mutational bias compared to the more fit, naturally occurring lines [28,29]. Second, some MA lines might go extinct–indeed, in most MA experiments they invariably do [7]. The extinct lines are likely to contain some of the most deleterious mutations that will be missed in the final sample of mutations; thus the sequencing of the surviving lines necessarily does not provide a fully unbiased sample of mutations.

An alternative approach to MA experiments relies on the identification of mutations from sequencing of genomes of natural strains. Unlike controlled laboratory experiments, such sequencing can be carried out with most species. Sampling from natural populations further removes many potential biases introduced by lab conditions and experimental set up. Methods that infer mutational spectra from sequence data usually rely upon the assumption that mutations at certain genomic locations are strictly neutral, such as pseudogenes or dead transposable elements [30] that are presumably under no selection pressure, or mutations that lead to a synonymous change in a protein-coding sequence. If this assumption holds, it can be shown that the rate of substitution between species at these sites would directly reflect variation in mutation rates [31–33]. However, it is increasingly apparent that almost no mutations are truly neutral, and even very mild selection or selection-like forces such as biased gene conversion can significantly influence patterns of substitution [34–38]. The overwhelming majority of substitutions observed from sequence data would therefore be survivors of selection and selection like forces, albeit to varying degrees. While extremely informative in their own right, these are necessarily highly biased subsets of the true spectrum of spontaneous mutations.

While divergence data are almost certainly biased by selection, existing polymorphisms within a population need not all be. Segregating alleles can be effectively neutral if they are observed while still under the selection-drift barrier. Because spontaneous mutations necessarily enter the population at a frequency of 1/N, where N is the number of the chromosomes in the population, identifying a cohort of extremely rare polymorphisms will enrich for very young mutations [39]. Mutational spectra from rare variants through deep population sequencing has already been employed in viral systems such as HIV [40], where the main challenge lies in accurately calling extremely rare variants from a heterogeneous viral population [41–43]. Rare variants have also been applied to characterizing context dependent mutational patterns in 202 human genes [44], although in species where single individual sequencing is accessible and populations are not homogeneous, population structure must be accounted for [45].

One elegant solution would be limiting analysis to *de novo* variants in parent offspring genome comparisons, such as the comparison of family trios in drosophila, butterfly, and humans [46–49]. In many other species, it is not always possible to identify relatedness between individuals ahead of time and selectively sequence parent-offspring genomes. In such instances the relatedness of sampled genomes or genomic regions must be estimated *post hoc*. For a hypothetical organism that reproduces asexually and does not undergo recombination, relatedness between individuals simply involves genomic sequence identity. If two genomes are nearly identical, any variant between them is likely a relatively young mutation that occurred after their last common ancestor. In actual datasets, recombination and/or sexual reproduction result in genomes with mosaic evolutionary history across genomic segments. To obtain recent mutations from such sequences, regions of identity by descent (IBD) would be more appropriate. However, proper IBD analysis requires haplotype information, which may not always be available, or might be difficult to impute in species such as yeast where ploidy can vary between 1n and 4n in natural isolates [50].

In the absence of IBD information, on the basis that rare polymorphisms are younger on average, the density of unique SNPs serves as a proxy for IBD information. Genomes with close relatives in the dataset share most of their polymorphisms with at least one other strain and carry few unique mutations, most of which will be young, while genomes with no close relatives share fewer polymorphisms and appear to carry an excessively large number of unique mutations (singletons), most of which will be old. The density of singletons in a genome or genomic region [51], as defined by all polymorphisms present in a sampled population, can serve as a measure of the age of rare variants on that genome.

To test the practicality and accuracy of this technique, we sequenced 141 individual strains of *Saccharomyces cerevisiae* to high genomic coverage and analyzed the mutational spectrum that could be obtained from identified young mutations. By comparing how closely our results matched both theoretical expectations and the mutational spectrum derived from a large-scale MA experiment in yeast, we determined that we could recapitulate the mutation spectrum of a species through broad population sequencing, that is, the sequencing of a large number of individuals.

## Results

To sample a set of non-experimental individuals from a relatively diverse population, we sequenced 141 *S*. *cerevisiae* strains in their natural ploidy states [52]. The majority of these strains were clinical isolates, with around a dozen well-studied commercial and lab strains. Because yeasts are known opportunistic pathogens, this set of strains likely represents the diversity in human-associated yeast populations. SNPs were only called in comparison to the reference sequence of S288C in non-repeat regions after meeting filter requirements (S1 Fig). Excluding one strain where sequencing failed due to contamination, a final set of 423,387 SNPs passed these quality filters (Methods). The site frequency spectrum of the observed population of polymorphisms shows the expected gamma shape of population sequencing datasets, with a small bump around freq = 1 (S2 Fig).

New spontaneous mutations, as a group, should show none of the classical signatures of selection. Three criteria were employed as indicators of our ability to identify very young SNPs: 1) the percentage of nonsynonymous polymorphism (%Pn), 2) the transition transversion (Ts/Tv) ratio, and 3) the GC equilibrium percentage (GCeqm). In divergence data, the ratio of nonsynonymous changes tends to be much lower than the ratio of 0.75 expected in the absence of selection, Ts/Tv values are usually > 2.5, and the GCeqm (roughly) matches the genomic GC content (which is 38% in yeast). The mutations from a previous large-scale genome-wide MA experiment in yeast yield a %Pn value close to the neutral expectation of 0.75, a Ts/Tv value of 1, and a GCeqm of 32% [12]. We therefore explored our ability to obtain similar values from our polymorphism data.

**Fig 1:**
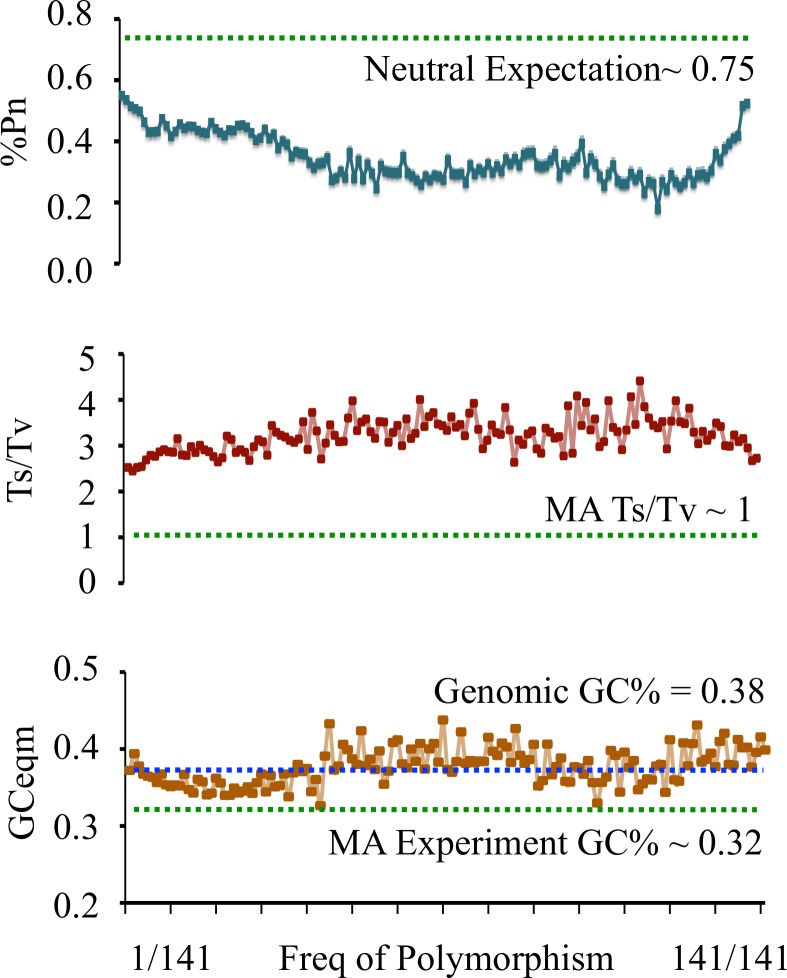
%Pn, Ts/Tv and GCeqm trends across SNP frequency. %Pn and Ts/Tv values show small shifts towards MA/neutral expectations in the lowest SNP frequencies (highlighted in box). X-axis–SNP frequency. Y-axis–%Pn, Ts/Tv, GCeqm.

We first segregated SNPs by their frequencies in the population and summarized all three values for each frequency class. We expected that with decreasing frequency of polymorphisms, the proportion of young SNPs should increase, and the three values should approach those observed in MA experiment (Fig 1 green dotted lines). While the %Pn and Ts/Tv ratios did shift towards MA values, especially in the lowest SNP frequencies, the changes did not reach expected MA values. However, a similar trend was not seen for the value of GCeqm (Fig 1). Indeed, even at the frequency of 1/141, none came close to matching MA values.

Because there is substantial population structure in the sampled strains [52] we tested whether controlling for relatedness between strains could further refine our analysis, this time focusing on just the singletons. We used the density of singletons/kb as a measure of singleton age. For example, if a chromosome carried *n* singletons, each of the *n* singletons is given the ‘age’ of *n*/length of the chromosome in kb, approximating the time unit it takes for a mutation to occur once per 1 kb since its last common ancestor with the closest sampled relative. Often, chromosomes will carry multiple singletons, and though the singleton mutations must have occurred at different times, it was impossible to accurately identify the order in which these mutations happened. We chose to be conservative in our age categorization and assign the same age to all singleton mutations on a given chromosome.

**Fig 2:**
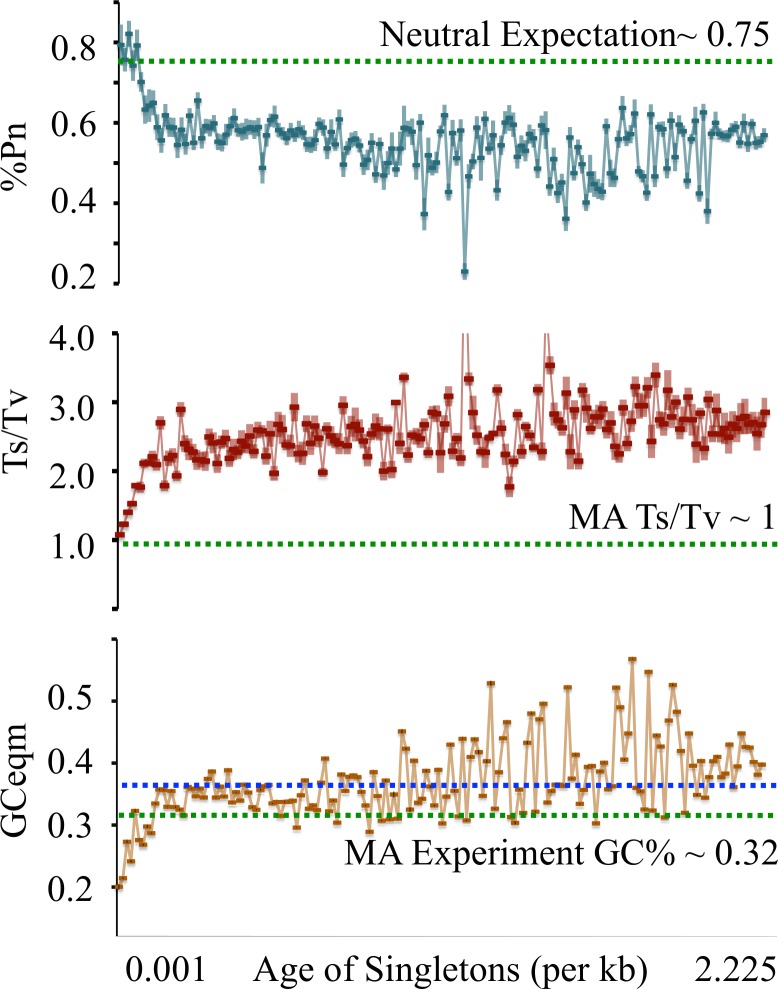
%Pn, Ts/Tv and GCeqm trends across SNP singleton age. %Pn and Ts/Tv values reach MA/neutral expectations in the lowest SNP frequencies (highlighted in box). GCeqm surpasses MA experimental values. X-axis–singletons from youngest to oldest. Y-axis–%Pn, Ts/Tv, GCeqm.

We binned SNPs by age into groups of roughly the same sample size, with higher resolution at the youngest ages, ranging from 0.001/kb through 2.25/kb. We then tested whether patterns derived from the younger age groups came closer to the MA experimental values. Plots of the %Pn, Ts/Tv, and GC equilibrium values for each age group showed a clear trend in which the 5 youngest categories (ages <0.005/kb) matched MA values for both Pn/Ps and Ts/Tv ratios (Fig 2). Surprisingly, for GCeqm, the youngest singleton classes suggested an average value of around 25%, below the 32% derived from the MA experiments (Fig 2). While mutations are indeed AT biased, this value is more extreme than previously reported. To ensure that the youngest singletons as a group were not dominated by low quality SNPs, we noted that coverage depth, genotype qualities, and mapping qualities were not significantly different between young singletons with density <0.005/kb as compared to older singletons, and SNP quality was capped at a minimum of 20 (S3 Fig).

**Fig 3:**
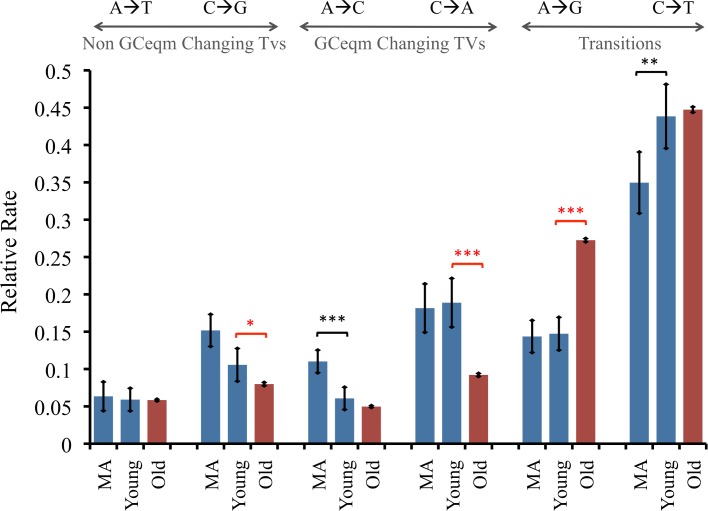
Relative mutation rates of the 6 nucleotide changes for MA experimental, young singletons, old singletons. Significant differences between rates in young singletons as compares to the other two datasets are annotated (Z-test, Bonferroni corrected). Error bars indicate S.E. * indicated *p*<0.05, ** indicate *p*<0.01, *** indicate *p*<0.001.

**Fig 4:**
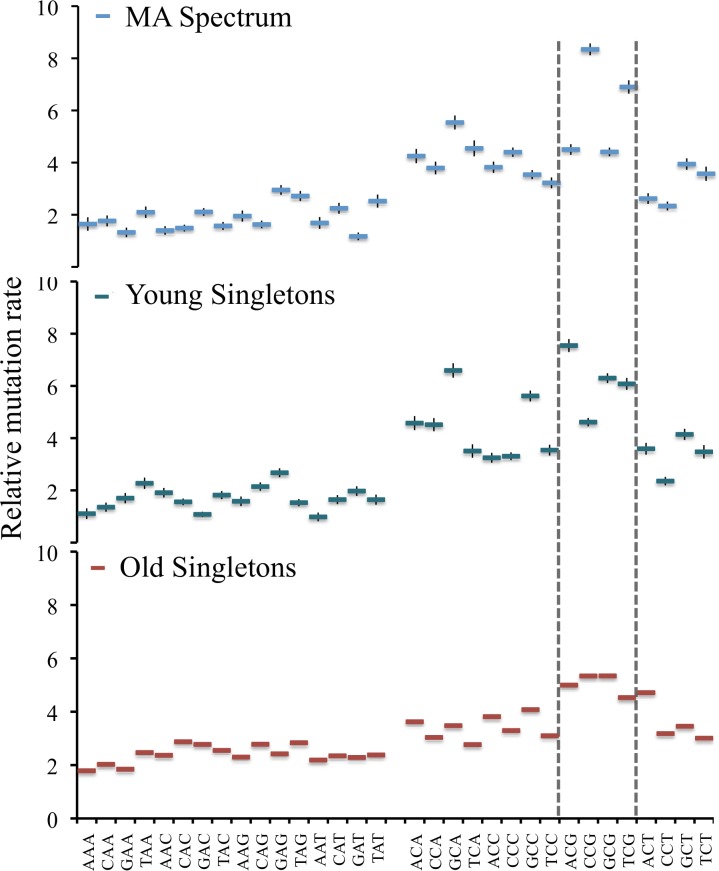
Neighbor dependent mutation rates in MA data, young singletons, and old singletons. X-axis–The 32 neighbor environments sorted. Y-axis–The relative rates in each environment.

There were 829 singletons of ages <0.005/kb that matched the Pn/Ps and Ts/Tv values from the MA experiment. Coincidentally, this sample size is similar to the 864 SNPs from the MA experiment. Because MA results were based on a single homozygous diploid strain that was exposed to a constant, stable environment, the mutation spectra of a population that is far less homogenous may be different. To determine how the mutation spectra presented by the young singletons differ from old singletons, or from MA data, we calculated the relative mutation rates for all six possible nucleotide changes (Fig 3). Young singleton rates for each nucleotide change were compared to corresponding old singletons and MA rates (Z-test, Bonferroni corrected). There were significant differences in rates between young singletons and old singletons, but also between young singletons and the MA mutations. We further pursued the context dependent difference in mutation rates previously found in MA data, and divided singletons into groups based on their neighboring bases. A previous MA experiment showed a potentially elevated mutation rate at the middle nucleotide C in CCG and TCG environments, suggestive of low but detectable levels of methylation [12]. However, this particular bias was not clearly observed in the young singletons. Indeed, the highest rate was observed at ACG sites in the young singletons. Intriguingly, all four *CG sites had higher mutation rates in the old singletons (Fig 4). The biological significance of these results remains to be determined as there is more recent evidence that there is in fact no methylation in *S*. *cerevisiae* [53]. Our results do suggest, however, that there might be subtle differences between MA estimates and mutational biases in nature. Additional data should be able to resolve this question.

**Fig 5:**
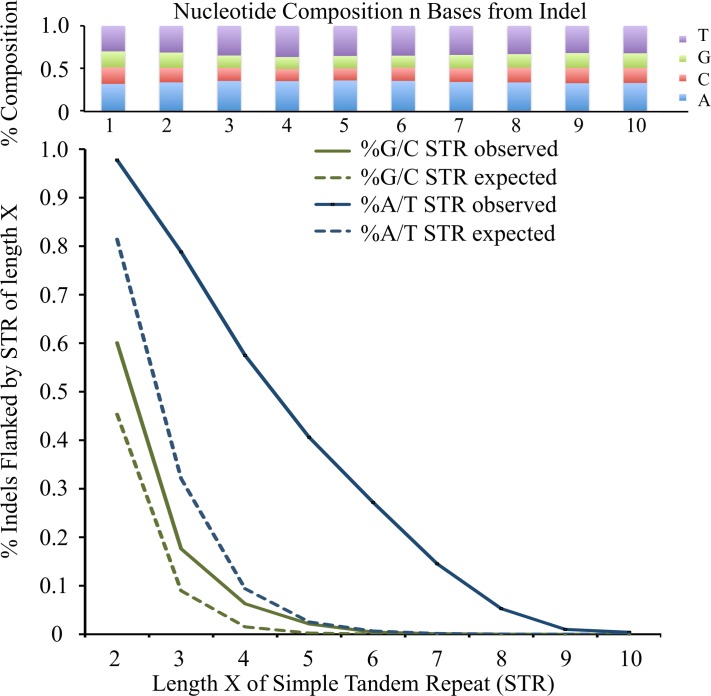
Top panel: base composition of ±10bp flanking singleton indel positions. X-axis–the ±1-10th base from indel position, 5’->3’ oriented. Y-axis–composition of A,C,G,T bases at that position across all indel flanking regions. Bottom panel: % of indels flanked by single base simple tandem repeats (STRs). X-axis–the length of monomer STR. Y-axis–the % of indels flanked by a monomer STR at least that long.

**Fig 6:**
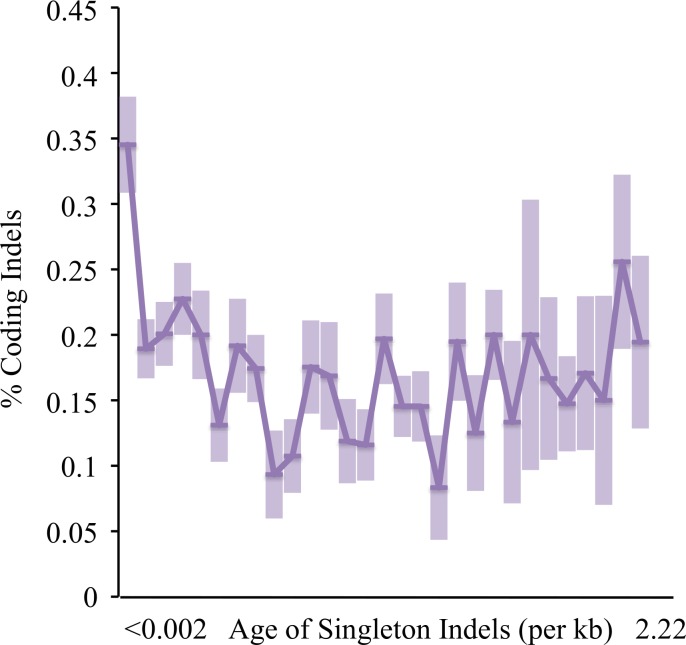
% of indels within coding sequences by age. X-axis–age of indel. Y-axis–percentage of indels found within coding regions.

We tested if this classification system could potentially be employed for another mutation class–indels. Indels have been more difficult to study and analyze than SNPs due to their exceedingly rare nature (observed at least an order of magnitude less often than SNPs) and their strong fitness effects (that do not usually allow them to persist in natural populations). In most MA experiments, indels are observed in very low numbers in unique sequences, particularly in coding regions. Broad population sequencing allows larger numbers of such events to be observed, but mapping errors can increase false discovery rate (FDR) around repetitive regions. We filtered and aged indels following the same protocol as SNPs, and utilized the percentage of indels seen within coding regions (which span ~70% of the analyzed portion of the yeast genome) as the main signature for the action of selection. We confirmed that GC content of genomic sequences ±10bp of 3,389 high quality singleton indels were not significantly biased, but the incidence of simple tandem repeats (STRs) were more common than expected by chance ±10bp of indels, particularly for A/T monomers (Fig 5). This is in spite of the prior masking of 600Mb of known repetitive sequences. The indel singletons also did not occur randomly within the genome, with only 20% found in coding sequences, although this may be partly due to context dependent variation in error rates [13,54–55]. However, the youngest indels of age <0.002/kb were clearly less constrained by selection than older indels (Fig 6).

## Discussion

Identifying young mutations when they first enter the population, and more critically before they have had a chance to rise above the selection drift barrier, is the equivalent of directly observing spontaneous mutations in a non-lab setting. Young mutations are necessarily rare, and can be captured through extremely broad population sequencing. To minimize noise from older alleles that appear rare simply due to biased sampling, linkage information can be leveraged. So far, mutational spectra deduced through extremely young alleles from broad population sequencing has not been cross-validated by MA experiments.

We sequenced 141 *S*. *cerevisiae* strains and were able to identify a subset of singletons that appeared to exhibit almost no signatures of selection, indicating their extremely young age. They also described a mutational spectrum similar to that previously detailed in a large-scale MA experiment, concurrently verifying the results from both techniques. However, the neighbor-dependent mutational trends appeared to vary across the datasets. While it is possible that neighbor dependency could vary across strains, this would require more data to clarify.

### Applications to other species

In yeast, we used singleton density per Kb of genomic sequence as the arbitrary genomic unit for singleton counts. Any genomic unit or segment can conceivably be used, as long as they are long enough such that mutations within that region are rare, but not so long that the sample does not contain individuals closely related enough as to be nearly identical across all of it.

It is also important to note that yeast has an atypical life cycle that is neither obligate asexual or sexual. It is thought to reproduce predominantly through clonal means with occasional sexual reproduction (reviewed in [56]). Yeast also has a marked tolerance for large-scale copy number changes (e.g. [57]). It may even be highly tolerant of hybridization with closely related species (e.g. [58–60]), and is known to carry introgression from sister species (e.g. [57]) as well as more distant relatives (e.g. [61]). The impact of such irregular life cycles (found in many fungi/moss species) on segregating sites within a population sample is not clear. A similar study in more species may help to resolve this question. In obligate sexual reproducers, there may still be large variations in mutation rate, recombination rate, or population diversity that can make sampling closely related genomes difficult without prior knowledge. For such species, more care must be taken during sample collection.

Another point to keep in mind is that this method can only identify what selection doesn’t immediately remove. There is a practical limit to how closely related individuals from a random population sampling can be, unless the population is extremely inbred, or there is genealogy information. The youngest mutation we can identify is consequently lower bound by how recently the two closest related individuals diverged. If selection is so strong that many mutations have already been removed within that short divergence time, we would be limited to only describing the trends that follow. An extreme example of such scenarios would be lethal mutations, although this was also seen to a certain degree in indels, which are removed by selection at a rate that is 10 times as fast as nonsynonymous mutations [19], and unsurprisingly never reached neutral expectations in our analysis.

### Advantages and limitations

The power of this method lies in numbers. Sequencing of just 141 strains was able to give us 829 putative young SNPs, a number nearly matching that from a large diploid yeast MA experiment involving nearly ~311,000 generations under controlled lab conditions [7]. A subset of these young mutations may have accumulated during lab propagation for DNA extraction, but the large numbers suggest that the majority were in fact ‘natural’ mutations. In addition, we identified 168 singleton indels with an age of <0.002/Kb, a class of mutation only very rarely seen in experimental settings. We were able to show that even here, where selection acts strongly and quickly to change the overall signature, some trends can still be observed with a decrease in indel age.

There are multiple benefits unique to this analysis. First, instead of correcting for population structure, an issue common to most population samples, it takes advantage of the varying degrees of relatedness in a sample set to classify singletons into age groups. These continuous age groups, in addition to investigating whether young mutations match neutral expectations, also allow observation of trends across time. Second, unlike methods dealing with divergence data, there are no phasing or haplotype issues. Young singletons are necessarily the derived allele, and they are so rare the effect of linkage is negligible. The yeast strains used were in fact in various states of natural ploidy [52], as can be expected of a natural population sample. However, note that <1% of sites were suspected of carrying more than 2 alleles, and were not considered in this analysis.

One major limitation of this method is that it doesn’t provide the ability to accurately estimate mutation rate, which is something that naturally follows from an MA experiment. The means of accurately estimating generation time separating such closely related individuals is beyond the scope of this manuscript. A second issue is that the number and identity of singletons will heavily depend upon which and how many strains are sequenced. As more strains are sequenced, some singletons will be lost, while others will be identified. A logical further extension of this approach would be to try to age not just singletons, but doubletons, tripletons etc., based on population frequency and shared haplotype lengths, though it is unclear how much this would modify the overall conclusions.

As broad population sequencing becomes increasingly accessible, the amount of information we can extract from resulting sequence data becomes the limiting factor to their scientific value. Well-described mutational spectra form one area of molecular evolution for which extensive work has been difficult to amass, and which can benefit from this new application.

## Materials and Methods

DNA library construction, read mapping, and variant calling protocol was detailed in earlier publication [52]. Briefly, DNA was extracted from liquid cultures using a modified glass bead lysis protocol. 500bp paired-end Illumina sequencing libraries were prepared at The Genome Institute, Washington University School of Medicine, and run on an Illumina HiSeq to an average of 100-fold coverage. Resulting fastq files were mapped to the reference genome with bwa v0.5.9 [62], sorted and indexed with samtools v0.1.18 [63], and assigned strain IDs with picard tools v1.55. Duplicated read pairs were removed and remaining reads locally realigned with GATK v2.1–8 [64]. The UnifiedGenotyper was used to call candidate variants across each sample independently. The resulting VCF files were filtered for variants with MQ>40, GQ>20, Qual>20, coverage depth >8X, >2 reads and >15% of reads supporting alternative variant. Around 600kb of the genome–annotated in the SGD database as simple repeats, centromeric regions, telomeric regions, or LTRs were excluded from analysis due to their susceptibility to mismapping and associated miscalls. Custom scripts were written to parse, identify, count, and summarize variants for every frequency, age, mutation type, and neighborhood category. Error bars were calculated as sampling errors where possible, or else estimated with 500 bootstraps.

## Supporting Information

S1 Fig Mapping quality across SNPs of varying frequencies. (TIF)

S2 Fig Site frequency spectrum of SNPs called from 141 MA strains (TIF)

S3 Fig Violin plots of mapping quality (MQ), read coverage (Cov), SNP quality (Qual), and genotype quality (GQ) for young singletons and old singletons. (TIF)
