# Supplementary figures and images for: Extremely Rare Polymorphisms in Saccharomyces cerevisiae Allow Inference of the Mutational Spectrum
Source: PLoS Genet. 2017 Jan 3;13(1):e1006455. doi: 10.1371/journal.pgen.1006455 (PMC5207638; doi:10.1371/journal.pgen.1006455)

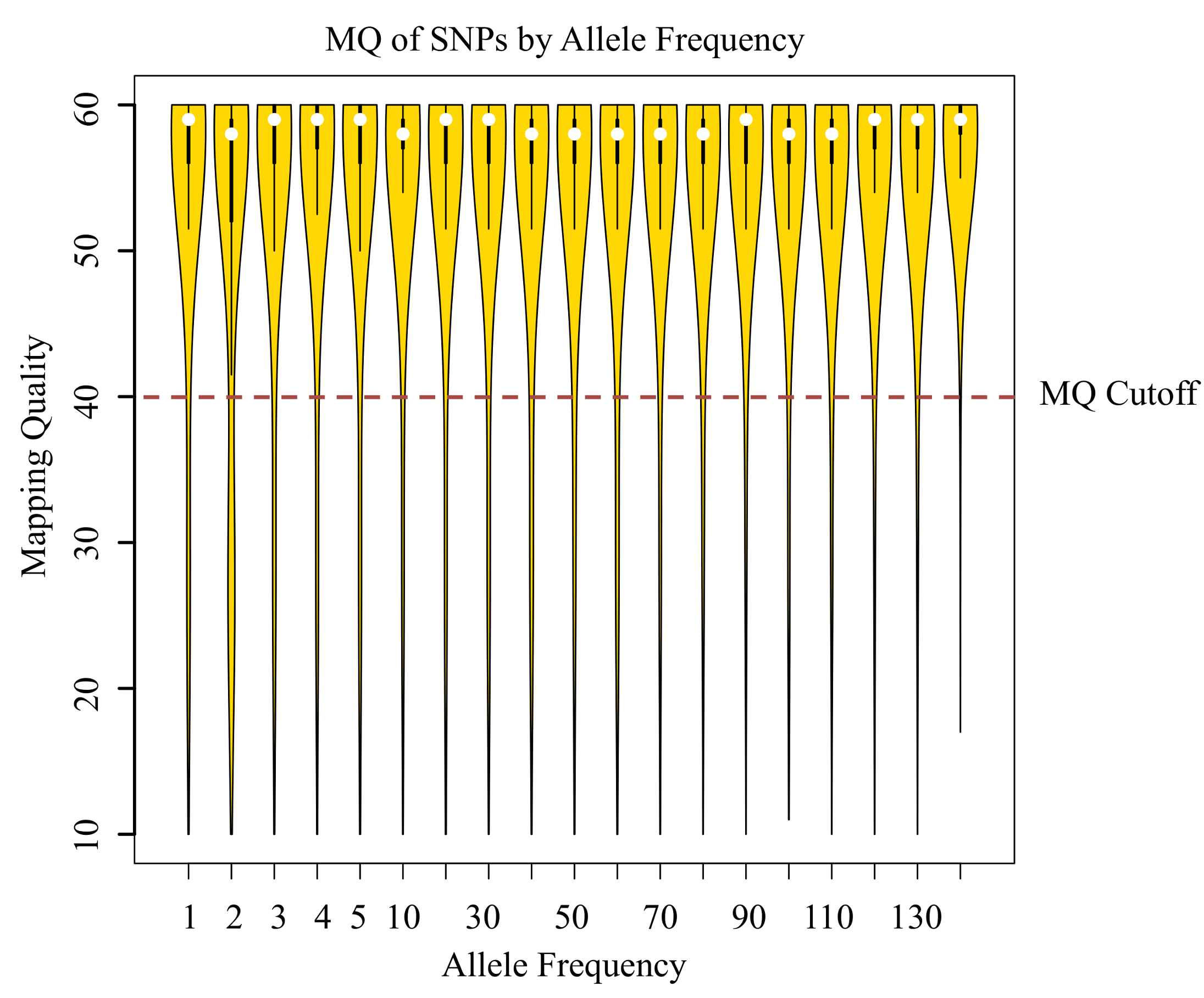

Supplement: S1 Fig — (TIF) [file pgen.1006455.s001.tif]

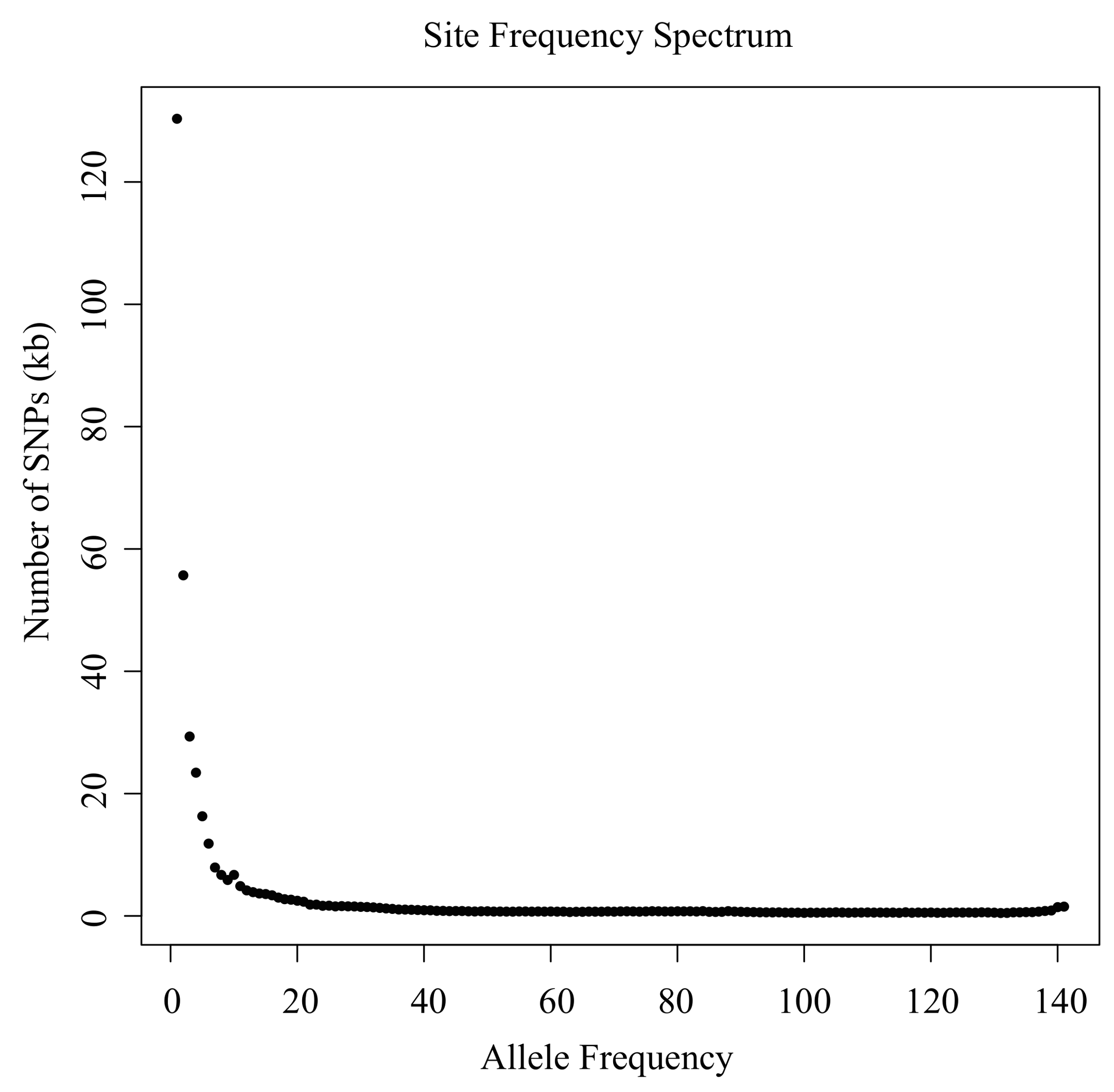

Supplement: S2 Fig — (TIF) [file pgen.1006455.s002.tif]

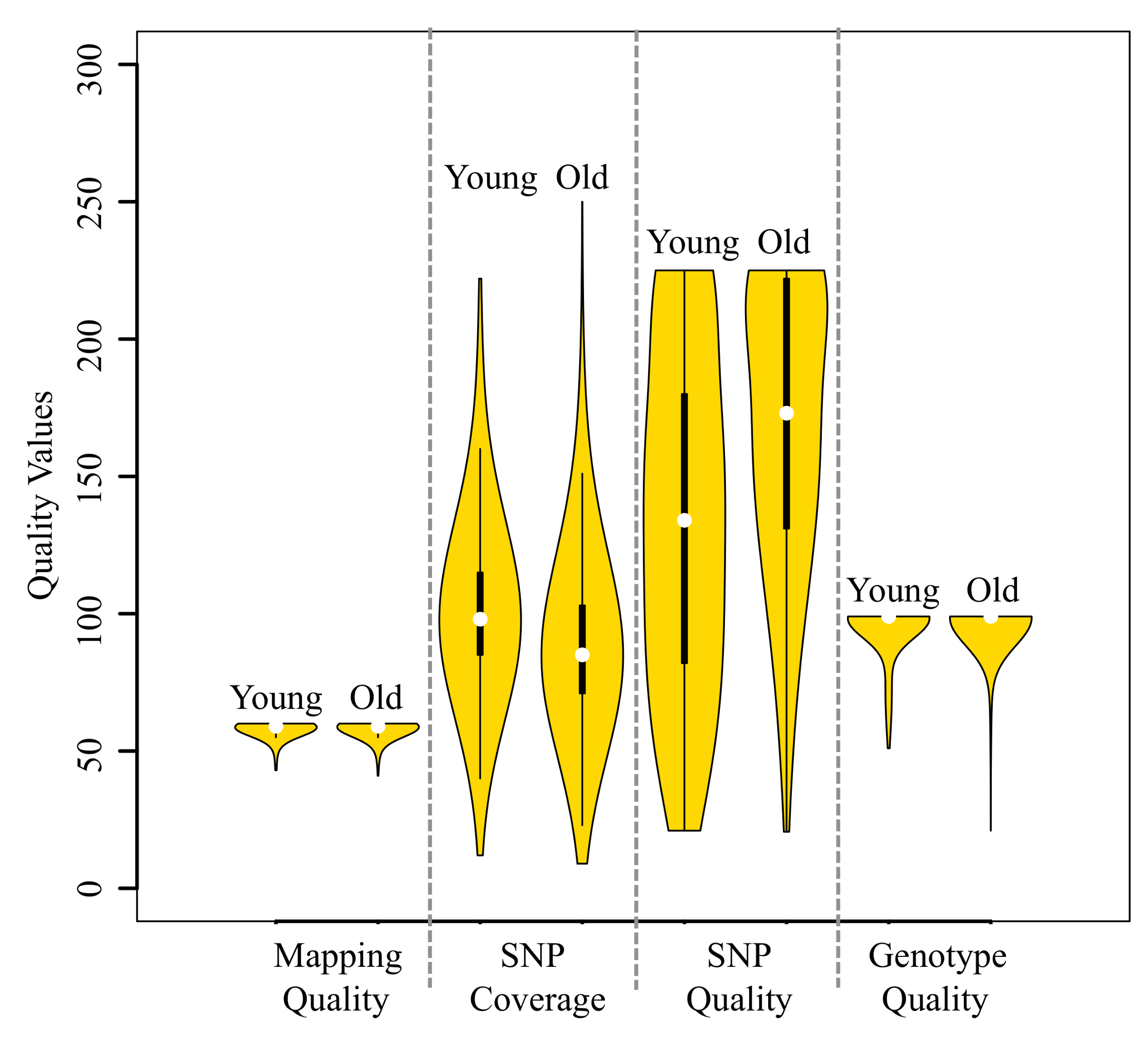

Supplement: S3 Fig — (TIF) [file pgen.1006455.s003.tif]
